# A possible connective tissue primary lymphoepithelioma-like carcinoma (LELC)

**DOI:** 10.3332/ecancer.2010.197

**Published:** 2010-12-22

**Authors:** G Aurilio, V Ricci, F De Vita, M Fasano, N Fazio, M Orditura, L Funicelli, G De Luca, D Iasevoli, F Iovino, F Ciardiello, G Conzo, F Nolè, MG Lamendola

**Affiliations:** 1 Medical Care Unit, Department of Medical Oncology; 4 Division of Medical Oncology; 8 Division of Radiology, European Institute of Oncology, Milan, Italy; 2 S Raffaele Scientific Institute, Medical Oncology, Milan, Italy; 3 Medical Oncology; 5 Pathological Anatomy Unit; 7 General Surgery Division, Second University of Naples, Naples, Italy; 6 PO Cardinale Ascalesi-ASL Na 1, Radiology, Naples, Italy

**Keywords:** lymphoepithelioma-like carcinoma (LELC), neck mass, lymphoepithelioma

## Abstract

Lymphoepithelial carcinoma is an undifferentiated nasopharyngeal carcinoma with lymphoid stroma and non-keratinizing squamous cells with distinctive clinical, epidemiological and etiological features. Conversely, lymphoepithelioma-like carcinomas (LELCs) are carcinomas that arise outside the nasopharynx but resemble a lymphoepithelioma histologically. In this case study, LELC presentation in connective tissue (left sternocleidomastoid muscle) is peculiar and unusual, but its diagnosis is supported by histological findings and clinical history, especially long disease free survival and no primary lesions in nasopharynx and lung district. We also discuss the pathogenesis, hypothesizing an embryological theory. To our knowledge, it could be the first reported case of a primary connective tissue LELC to the neck.

A 61-year-old man, without comorbidities, presented with a swelling on the neck associated with occasional pain. Neck ultrasound and computed tomography (CT) scan showed a 6x3 cm solid expansive mass to the soft tissue, on the left side of the neck, contiguous to the omolateral sternocleidomastoid muscle. This neoformation appeared non-homogeneous with irregular margins and extended from the plane passing through the hyoid bone to the left supraclavicular region with compression of the thyroid left lobe. Enlarged lymph nodes were present on both sides of the neck (Figure 1).

The patient underwent surgical removal of the mass, with sternocleidomastoid muscle, internal jugular vein and lymph node dissection on the same side. On macroscopic examination, lymph nodes of different sizes to the lower pole and a muscle section to the higher pole were recognizable. On cut, a 5-cm grey, compact area, tending to friability, within the muscle, was examined.

Histology showed malignant neoplastic tissue invading the striated muscle. The tumour was formed of irregular nests and trabeculae of large polygonal cells with large nuclei, clumpy chromatin and prominent nucleoli and syncytial appearance. The nests of epithelial tumour cells were associated with a dense and accentuated lymphoid infiltrate (Figure 2).

No keratinization could be seen in the tumour. Immunohistochemical examination revealed strong immunoreactivity for cytokeratins and epithelial membrane antigen (EMA) (Figure 3), and negativity for vimentin, CD3 and CD20 (Figure 4).

Conversely, the lymphocytic infiltrate was intensely stained with CD45 (leucocyte common antigen—LCA). Using subset markers, the large amounts of lymphocytes around the cords of carcinoma cells were CD3—positive T lymphocytes and CD20—positive B lymphocytes with a clear predominance of CD3—positive T lymphocytes, which consisted predominantly of CD8—positive cells > CD4—positive cells. Some cells were also positive for lysozime and CD68 (macrophages).

*In situ* hybridization (RNA-ISH) and polymerase chain reaction (PCR) for detection of Epstein–Barr Virus (EBV) genome were negative in both epithelial and lymphocytic populations.

Lymph nodes were extensively necrotic and metastatic.

Morphological and immunohistochemical findings fulfilled the criteria for the diagnosis of lymphoepithelioma-like carcinoma (LELC).

A rhinofibrolaringoscopy and head-and-neck magnetic resonance imaging (MRI) did not show any residual tumour mass.

The patient was followed with CT-scan and MRI, and approximately four years after surgery is currently disease free.

Lymphoepithelial carcinoma, also known as lymphoepithelioma or nasopharyngeal carcinoma type III or Schmincke–Regaud tumour, is an undifferentiated nasopharyngeal carcinoma with lymphoid stroma and non-keratinizing squamous cells with distinctive clinical, epidemiological and etiological features [1].

Lymphoepithelioma-like carcinoma is a carcinoma arising outside of the nasopharynx, resembling lymphoepithelioma both histologically and immunophenotypically. It is a malignant epithelial tumour which is characterized by large and uniform cells with indistinct cytoplasmic borders, resulting in a syncytial growth pattern, round to vescicular and ovoid nuclei, and large central nucleoli [2]. Usually, an inflammatory infiltrate rich in mature lymphocytes (predominantly CD8+ and T-cells) and occasionally eosinophiles are also present, partially obscuring the neoplastic epithelial component [3].

Conventionally, LELCs have been classified as ‘high grade’ based on poor histological differentiation. LELC localizations have been described to the gastrointestinal tract [4], thymus, salivary glands [5], uterine cervix [6], skin [7], lung [2], oral cavity [8], breast [9], vagina [10], trachea, larynx and vocal cord [11], bladder, rarely to the renal pelvis, ureter [12], and only once to a scar [13].

While there is a strong etiopathological association between EBV and lymphoepithelioma, mostly in South-Eastern Asian people [14], a connection between LELC and EBV is more variable, and usually without any ethnic relevance. However, in cases of LELCs occurring in pharyngeal or foregut derivatives, the association is much stronger in Asians than in Caucasians [2]. Recently, EBV has consistently been observed in LELC of specified anatomic sites, such as lung, stomach, thymus and salivary gland [5].

Circulating serum EBV DNA could be used as a tumour marker in the clinical management of LELCs. In fact, in published results, patients with a pre-therapy serum EBV DNA > 10.000 copies/ml had significantly worse survival [15]. Elevated levels six–eight weeks after therapy were strongly associated with both progression-free and overall survival [16].

At present, no validated therapeutic management exists for LELC. Surgery represents the best option in localized disease [5] sometimes followed with radiotherapy [17], whereas chemotherapy is not a conventional option.

Taxanes, platinum derivatives and 5-fluorouracil combined with folinic acid are active drugs [18].

The LELC has generally been considered to be highly radiosensitive, and post-operatory radiotherapy is indicated in cases of residual neoplastic tissue at the surgical margins, high-grade tumour, tumoural infiltration of adjacent organs or extensive locoregional diffusion [19, 5].

In locally advanced stages, a multimodality approach, including surgery, radiotherapy and chemotherapy, is advised [20].

Prognosis is linked to stage, grading, radical surgery and radiotherapy when the resection is not complete [5].

In the differential diagnosis of a connective tissue mass within the neck region an LELC should be considered as well as nasopharyngeal and metastatic squamous-cell lung carcinomas. Histologically, LELC cannot be distinguished from them, but clinical history and absence of a primary lesion to the nasopharynx or lung could drive the diagnosis.

Furthermore, a clear distinction between non-keratinizing carcinoma and large-cell lymphoma can be difficult. Accurate diagnosis is aided by a panel of immunohistochemical stains that uses epithelial markers and common leukocyte antigens.

In the present report, features favouring diagnosis of carcinoma include the presence of cohesive cell groups with poorly defined cell borders and an immunohistochemical profile of the neoplastic cells showing strong positivity for cytokeratin and EMA but negativity for LCA.

This presentation in a connective tissue appears to be very peculiar and unusual. To our knowledge, it could be the first reported case.

In our case, a possible primary site was not found, even though it cannot be absolutely excluded. However, a primary connective tissue LELC could be assumed, based on the branchial embryonic remnants origin, particularly from branchial cysts arisen from the second branchial arch [21]. In fact, these cysts, consisting of lymphoid aggregates that can have either squamous or respiratory-type epithelial lining, occur mostly along the front edge of the sternocleidomastoid muscle from the plane passing through the hyoid bone to the suprasternal notch [21, 22, 23].

These branchial cysts may develop into branchiogenic carcinomas starting from the cystic epithelial lining, which is considered a branchial remnant situated along the front edge of the sternocleidomastoid muscle, not associated with the development of any cancer within five years after diagnosis [24].

Finally, the long disease free survival, uncommon for a nasopharyngeal carcinoma, is another significant aspect suggesting a diagnosis of LELC.

## Figures and Tables

**Figure 1:**
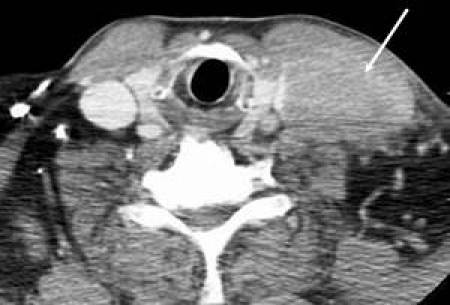
Axial CT scan of the neck showing a left supraclavicular mass that takes contact with the left jugular vein (narrowed and infiltrated) and the left sternocleidomastoid muscle.

**Figure 2:**
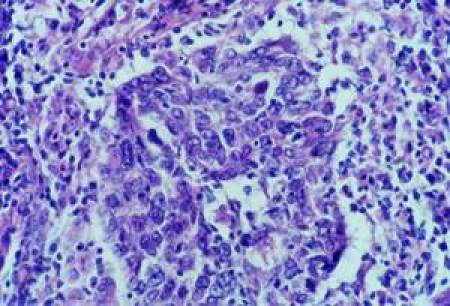
Cytology.

**Figure 3:**
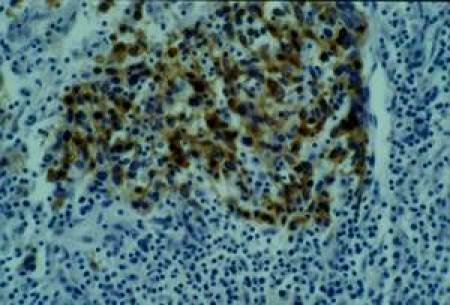
Cytokeratin and EMA immunohistochemistry positive.

**Figure 4:**
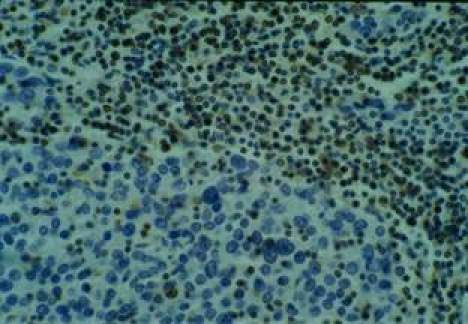
Vimentin and lymphoid markers immunohistochemistry negative.

## References

[1] Dohnert G (1971). Lymphoepithelioma Schmincke-Regaud. Virchows Arch A Pathol Pathol Anat.

[2] Bildirici K, Ak G, Peker B, Metintaş M, Alataş F, Erginel S, Uçgun I (2005). Primay lymphoepithelioma-like carcinoma of the lung. Tuberk Toraks.

[3] Kobayashi M, Ito M, Sano K, Honda T, Nakayama J (2004). Pulmonary lymphoepithelioma-like carcinoma: predominant infiltration of tumor-associated cytotoxic T lymphocytes might represent the enhanced tumor immunity. Intern Med.

[4] Chen PC, Pan CC, Hsu WH, Ka HJ, Yang AH (2003). Epstein-Barr virus-associated lymphoepithelioma-like carcinoma of the esophagus. Hum Pathol.

[5] Sun XN, Xu J, Yang QC, Hu JB, Wang Q (2006). Lymphoepithelioma-like carcinoma of the submandibular salivary gland: a case report. Chin Med J (Engl).

[6] Bais AG, Kooi S, Teune TM, Ewing PC, Ansink AC (2005). Lymphoepithelioma-like carcinoma of the uterine cervix: absence of Epstein-Barr virus, but presence of a multiple human papillomavirus infection. Gynecol Oncol.

[7] Cavalieri S, Feliciani C, Massi G, Addolorato G, Gasbarrini G, Amerio P, Rotoli M (2007). Lymphoepithelioma-like carcinoma of the skin. Int J Immunopathol Pharmacol.

[8] Chow TL, Chow TK, Lui YH, Sze WM, Yuen NW, Kwok SP (2002). Lymphoepithelioma-like carcinoma of oral cavity: report of three cases and literature review. Int J Oral Maxillofac Surg.

[9] Peştereli HE, Erdogan O, Kaya R, Karaveli FS (2002). Lymphoepithelioma-like carcinoma of the breast. APMIS.

[10] McCluggage WG (2001). Lymphoepithelioma-like carcinoma of the vagina. J Clin Pathol.

[11] Sone M, Nakashima T, Nagasaka T, Itoh A, Yanagita N (1998). Lymphoepithelioma-like carcinoma of the larynx associated with an Epstein-Barr viral infection. Otolaryngol Head Neck Surg.

[12] Yamada Y, Fujimura T, Yamaguchi T, Nishimatsu H, Hirano Y, Kawamura T, Teshima S, Takeuchi T, Kitamura T (2007). Lymphoepithelioma-like carcinoma of the renal pelvis. Int J Urol.

[13] Lyle P, Nakamura K, Togerson S (2008). Lymphoepithelioma-like carcinoma arising in the scar from a previously excised basal cell carcinoma. J Cutan Pathol.

[14] Iezzoni JC, Gaffey MJ, Weiss LM (1995). The role of Epstein-Barr virus in lymphoepithelioma-like carcinomas. Am J Clin Pathol.

[15] Ngan RK, Yip TT, Cheng WW, Chan JK, Cho WC, Ma VW, Wan KK, Au JS, Law CK (2004). Clinical role of circulating Epstein-Barr virus DNA as a tumor marker in lymphoepitelioma-like carcinoma of the lung. Ann N Y Acad Sci.

[16] Griffin BE, Xue SA (1998). Epstein-Barr virus infections and their association with human malignancies: some key questions. Ann Med.

[17] Han AJ, Xiong M, Gu YY, Lin SX, Xiong M (2001). Lymphoepithelioma-like carcinoma of the lung with a better prognosis. Am J Clin Pathol.

[18] Chan AT, Teo PM, Lam KC, Chan WY, Chow JH, Yim AP, Mok TS, Kwan WH, Leung TW, Johnson PJ (1998). Multimodality treatment of primary lymphoepithelioma-like carcinoma of the lung. Cancer.

[19] Ho JC, Lam WK, Ooi GC, Lam B, Tsang KW (2000). Chemoradiotherapy for advanced lymphoepithelioma-like carcinoma of the lung. Respir Med.

[20] Dubey P, Ha CS, Ang KK, El-Naggar AK, Knapp C, Byers RM (1998). Non-nasopharyngeal lymphoepithelioma of the head and neck. Cancer.

[21] Ayache D, Ducroz V, Roger G, Garabédian EN (1997). Midline cervical cleft. Int J Pediatr Otorhinolaryngol.

[22] Shvero J, Hadar T, Avidor I, Abraham A, Sidi J (1986). Heterotopic salivary tissue and branchial sinuses. J Laryngol Otol.

[23] Regauer S, Gogg-Kamerer M, Braun H, Beham A (1997). Lateral neck cyst the branchial theory revisited. A critical review and clinicopathological study of 97 cases with special emphasis on cytokeratin expression. APMIS.

[24] Martin H, Morfit HM, Ehrlich H (1950). The case for branchiogenic cancer (malignant branchioma). Ann Surg.

